# Lung Function and Respiratory Morbidity Among Informal Workers Exposed to Cement Dust: A Comparative Cross-Sectional Study

**DOI:** 10.5334/aogh.4089

**Published:** 2023-07-04

**Authors:** Kumar Dushyant, Gagandeep Kaur Walia, Niveditha Devasenapathy

**Affiliations:** 1 Indian Institute of Public Health-Delhi, Public Health Foundation of India, IN; 2 Public Health Foundation of India, IN

**Keywords:** Occupational health, informal workers, cement dust, lung function, India

## Abstract

**Background:**

Cement dust is a significant source of occupational exposure affecting lung function and respiratory health. A higher burden of respiratory morbidity is known among factory workers involved in cement production. Globally or from India, there are no estimates of this burden from informal workers exposed to cement dust.

**Objective:**

To assess difference in lung function and respiratory symptoms among informal workers exposed to cement and those unexposed, using a comparative community based cross-sectional study from purposively selected areas in Delhi, India.

**Methods:**

Using a portable spirometer we measured lung function and collected respiratory symptoms from conveniently sampled informal workers (n = 100) exposed to cement dust, 50 indoor informal workers (tailors), and 50 outdoor (vegetable) vendors. Regression analyses were performed to compare respiratory symptom score and lung function parameters, adjusted for age, body mass index, smoking, socioeconomic status, and years of occupational exposure.

**Findings:**

Exposed workers had significantly lower lung function (PEF = –750 ml/s and –810 ml/s and FEV1/FVC (%) = –3.87 and –2.11) compared to indoor and outdoor groups, with three times higher chronic respiratory symptoms when compared to the unexposed groups. The cement dust exposure was observed to be associated with PEF (mean difference (MD) = –0.75L, 95%CI = –1.36 to –0.15, p = 0.01), %FEV1/FVC (MD = –3.87, 95%CI = –6.77 to –0.96, p = 0.03) and respiratory symptoms (p < 0.001).

**Conclusion:**

This study generates evidence regarding the respiratory burden of occupational exposure among vulnerable informal workers. There is an urgent need for policy reforms to safeguard health from occupational exposures, especially among informal workers.

## Introduction

Chronic respiratory diseases contribute 10.9% of the total deaths in India, of which chronic obstructive respiratory diseases (COPD) and asthma contribute 8.7% and 1.9%, respectively [1]. Of the total global DALYs (Disability adjusted life years), 3.9% was due to chronic respiratory diseases, of which India alone contributes 32% [2]. There are several occupational exposures that are known to cause respiratory morbidity and mortality [3]. Cement dust is a common and well known harmful exposure among individuals involved in manufacturing of cement and/or engaged at construction sites. The respirable crystalline silica particles in cement dust are the main reason for lung damage [4]. Observational studies have shown lower lung functions (Forced Vital Capacity (FVC): –200 ml to –1200 ml; Forced Expiratory Volume in 1second (FEV1): –300 ml to –800 ml; Peak Expiratory Flow Rate (PEFR) –1 L/s to –2.5 L/s [5,6,7,8,9,10,11,12] and higher respiratory morbidity among factory workers with chronic exposure to cement dust compared to those less exposed or not exposed [5,7,8,9,11,12,13,14].

In India, cement factories are registered under the 1948 Factories Act that ensures safety and health of the workers in formal sectors [15]. It is well known that 92% of employment in India is contributed by informal workers and that they are not under any safety act [16]. Notably, there is a significant contribution by the informal sector in the journey of cement from factories to the site of construction. There are currently no data documenting the lung function status or respiratory morbidity among informal workers chronically exposed to cement. Most informal workers are immigrants from poorer neighboring states and they have the highest proportion of extreme poverty compared to self-employed or salaried households (18% versus 7%) [16]. This vulnerable population is generally not included in formal population-based surveys or factory-based studies, which may lead to underestimation of the overall burden of respiratory illnesses that is attributed to occupational exposures [17].

Thus, a cross-sectional study was conducted among informal cement workers in the national capital, Delhi. The objectives of this study were, (1) to compare the lung functions of informal workers exposed to cement dust with those not exposed to cement dust, (2) to compare the prevalence of self-reported respiratory symptoms between the exposed and unexposed groups.

## Methods

This was a comparative cross-sectional study and we followed Strengthening the Reporting of Observational studies in Epidemiology (STROBE) guidelines to report our findings [18].

### Study Setting

The study was conducted in purposively chosen areas, Shakurbasti in Northwest Delhi and Tagore Garden in West Delhi from December 2018 to January 2019. All study participants were male informal workers belonging to three occupational groups; one group was exposed to cement dust and the other two groups were not. The Shakurbasti railways complex engages thousands of informal workers in loading and unloading of cement all through the year. These constituted the sampling frame of the exposed group. These migrant workers are mostly from the neighboring states and work in day and night shifts for 4–16 hours per day. Within one kilometer distance from the railway complex at Shakurbasti, indoor unexposed workers were sampled from a small-scale jeans factory. Tagore garden, situated 4 kms from the railway complex, has a parking lot of manual cycle rickshaws. Here, the second group of un-exposed informal workers (outdoor un-exposed group), primarily involved in selling vegetables on their cycle rickshaws, were sampled.

### Study Population

All voluntarily consenting individuals who could understand the regional language (*Hindi)* were included. The study protocol was approved by the institutional ethics committee of Indian Institute of Public Health, Delhi (IIPHD_IEC_S_27_2018). Written informed consent was obtained from each individual before administration of the questionnaire.

#### Sample Size

To detect a moderate effect (0.40) in lung function parameters between the exposure groups with 80% power and overall alpha set at 5% (no multiplicity correction), 100 individuals per group would be required. A mean difference of 0.2L [10] in FVC, with a SD of 0.5L (equal SD in each group was assumed), was assumed for the sample size estimation. However, due to feasibility issues, 100 participants in exposed and 50 participants each in indoor and outdoor unexposed were enrolled.

#### Sampling

At the individual sampling level, a convenience sampling method was adopted over random sampling since it was not feasible to have list of workers from the contractors who employed them. At each of the areas (the railway complex, the small-scale factory, and the rickshaw parking lot), camps were organized. Those attending the camp and were found to be eligible were included in the study.

### Data Collection

We captured socio-demographic characteristics, occupational details, type of cooking fuel, tobacco use, alcohol use, and any current or past illness using a structured pilot-tested questionnaire. Respiratory symptoms were collected using the respiratory questionnaire of the British Medical Research Council [19]. Lung function was assessed using a portable spirometer (NDD EasyOne WorldSpirometer (https://www.ndd.ch). Height (portable plastic Tanita Leicester Stadiometer), weight (digital Tanita weighing machine HS-301) and blood pressure (digital instrument Omron HIT HEM 7300) were measured using standardized procedures by the same field investigator. Data collected in paper forms were entered into Microsoft excel sheets.

#### Exposure and Outcome Assessments

The occupational exposure groups were the proxy measure for primary exposure of interest. Quantitative assessment of dose of exposure was not done. The exposed group was exposed to cement and outdoor air pollution. The outdoor comparison group was not directly exposed to cement but exposed to outdoor pollution and the indoor comparison group was not exposed to cement dust and spent most of the time indoors only.

Objective assessment of lung functions and self-reported respiratory symptoms were the key outcome variables. To measure the lung function parameters (FVC, FEV1 and PEFR), the participant was seated in a comfortable position and asked to take a long deep breath to the maximum extent. The mouthpiece of the spirometer was inserted, following which he was asked to expire forcefully and completely. A minimum of three such blows were recorded. Recording with differences within 0.15 L between the two highest recordings of FEV1 and FVC were considered as a valid measurement. If necessary, more blows, up to 8, were recorded. An FEV1/FVC (%) ratio less than 70% was considered suggestive of persistent air flow limitation [20]. Predicted values for FEV1 and FVC were automatically estimated in NDD software (https://www.ndd.ch) on the basis of age, height, weight, and ethnicity of the participant. FEV1% predicted was calculated and values lesser than 80 was considered as obstructive lung function and greater than and equal to 80 as normal lung function [20]. The respiratory questionnaire did not have a formal scoring system available; hence summary measures were estimated as described in the supplementary material. The purpose of this scoring was to differentiate between any respiratory symptoms and high-grade symptoms.

#### Description of Confounders

Socio-economic status (SES) was an un-weighted composite score (ranging from 0–9) using binary variables such as able to read or write, income more than 10,000 INR, LPG use, ownership of cycle, television, cable network, motorcycle, and mobile phone, and expenditure on phone (less or more than the average of overall sample mean). Other confounders were body mass index (BMI) in Kg/m^2^, age in completed years, years of work in the current occupation, and smoking status (never, former, and current smoker).

#### Statistical Methods

We present descriptive information of participant profile, lung function, and respiratory symptoms by exposure groups. Continuous variables are presented as mean, SD, or median, IQR based on their distributional properties, and categorical variables presented as frequencies and percentages. Using multivariable linear regression, we report mean differences in lung function along with 95% CI parameters adjusted for confounders. Group comparisons of respiratory symptom outcomes (any symptoms and any high-grade symptoms) were analyzed using multivariable logistic regression, and adjusted ORs with 95% CI are presented. Respiratory symptom score was compared using zero-inflated negative binomial regression to account for over-dispersion and excess zeros and we report rate ratios along with 95% CI. All analyses were adjusted for age, BMI, smoking status, SES, and years of work experience. The indoor group was considered as baseline group for all comparisons and P < 0.05 was considered as statistically significant. Assumptions of linearity of confounders, multi-collinearity of covariates, and normality of residuals were checked after regression analyses. Finally, correlation between respiratory symptom score and lung function parameters were assessed using Pearson’s correlation coefficient. All analyses were performed using Stata 14 [21].

## Results

**Figure 1:**
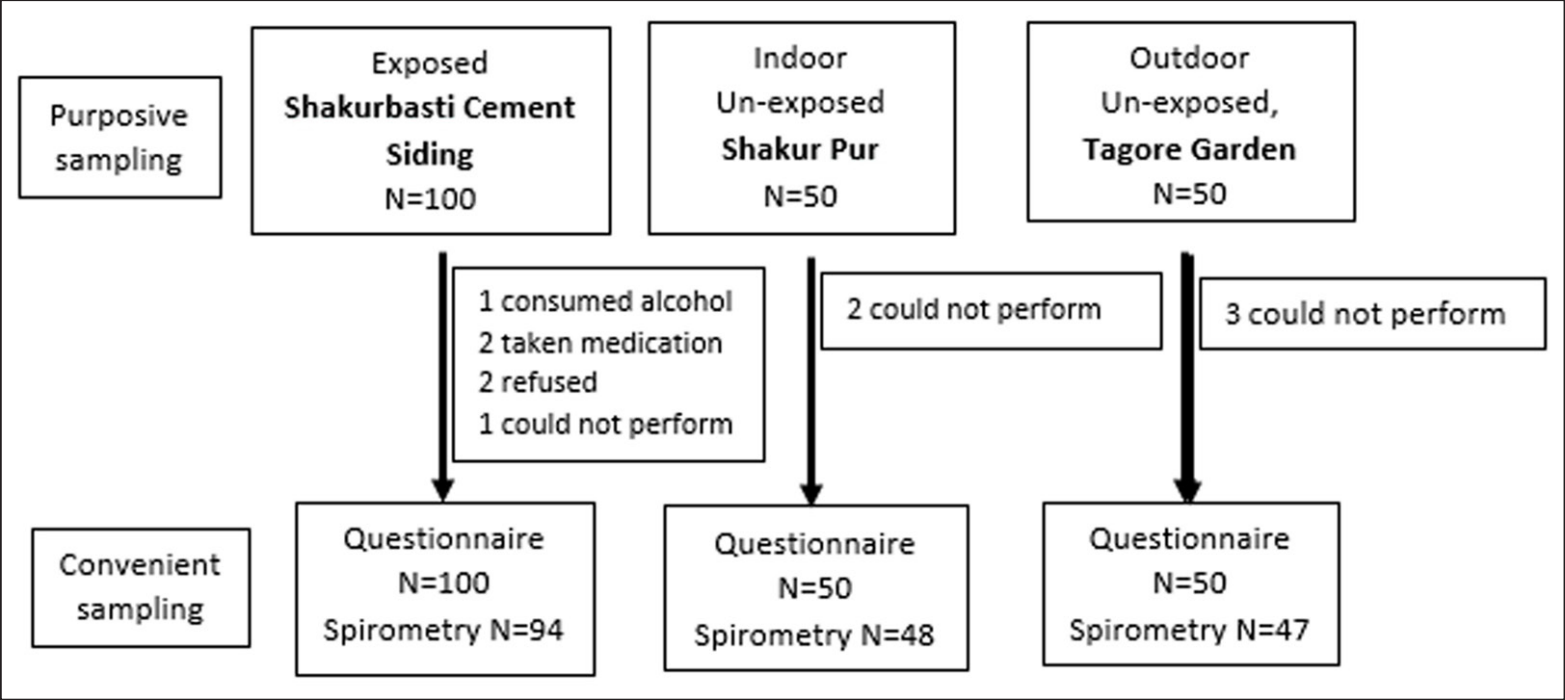
Sampling Scheme.

Two hundred participants provided information on socio-demography, lifestyle, and respiratory morbidity, of which 189 could provide valid spirometry readings (Figure 1). When compared to the combined (indoor and outdoor) unexposed group, exposed participants were older (40 years vs. 31 years), the majority were from a Muslim community (71% vs. 57%), literacy levels were low (43% vs. 79%), participants were living in *kuccha* houses (97% vs. 2%), and they had lower mean SES scores (3.18 vs. 4.26). Table 1 provides further information on demographic profile categorized by the three study groups.

**Table 1:** Socio-Demographic Characteristics Between Three Study Groups.


DEMOGRAPHY	CEMENT-EXPOSED N = 100	INDOOR UNEXPOSED N = 50	OUTDOOR UNEXPOSED N = 50

Age in years (Mean, SD)	40.1 (11.4)	33.9 (12.1)	33.6 (10.9)

Religion, *n (%)*			

Muslim	71 (71)	36 (72)	21 (42)

Non-Muslim	29 (29)	14 (28)	29 (58)

Caste, *n (%)*			

Non-general	24 (24)	22 (44)	35 (70)

General	64 (64)	28 (56)	15 (30)

Do not want to tell	12 (12)	0	0

Education, *n (%)*			

No schooling	62 (62)	13 (26)	17 (34)

Primary	22 (22)	19 (38)	16 (32)

Middle & Senior secondary	15 (15)	15 (30)	16 (32)

Graduate and above	1 (1)	3 (6)	1 (2)

Literacy (Read or Write)	43 (43)	43 (86)	36 (72)

Married, *n (%)*	96 (96)	37 (74)	39 (78)

Living with Spouse	28 (29.2)	12 (32.4)	17 (43.6)

Living with, *n (%)*			

Alone	33 (33)	6 (12)	5 (10)

Co-workers	43 (43)	28 (56)	20 (40)

Family only	23 (23)	16 (32)	24 (48

Family & other co-workers	1 (1)	0	1 (2)

Monthly income, *n (%)*			

<=10000 ₹	70 (70)	31 (62)	29 (58)

10001₹–25000₹	30 (30)	18 (36)	21 (42)

Missing	0	1 (2)	0

Household size (Median/IQR)	4 (1,5)	4.5 (3,5)	4 (3,6)

Electricity, *n (%)*	91 (91)	50 (100)	49 (98)

Metered, *n (%)*	7 (7.7)	48 (96)	49 (100)

Type of household, *n (%)*			

Kuccha	97 (97)	1 (2)	1 (2)

Semi Pucca	0	0	3 (6)

Pucca	3 (3)	49 (98)	46 (92)

Running water inside house, *n (%)*	5 (5)	47 (94)	45 (90)

Latrine, *n (%)*			

Open fields	4 (4)	0	0

Public place	89 (89)	0	1 (2)

Common	4 (4)	35 (70)	36 (72)

Inside house	3 (3)	15 (30)	13 (26)

Cooking done, *n (%)*			

Outdoors	3 (3)	1 (2)	2 (4)

Inside house	92 (92)	47 (94)	47 (94)

In a separate building	5 (5)	2 (4)	1 (2)

Fuel used for cooking, *n (%)*			

Bio	18 (18)	0	1 (2)

LPG	82 (82)	50 (100)	49 (98)

SES score* (Mean, SD)	3.18 (1.43)	4 (1.43)	4.52 (1.94)

BMI (Mean, SD)	23.2 (3.1)	22.8 (3.9)	22.9 (3.2)

Blood pressure (mm/hg)			

Systolic (Mean, SD)	138.6 (16.9)	140.5 (20.7)	137.2 (17.4)

Diastolic (Mean, SD)	85.5 (12.1)	83.3 (13.2)	81 (11.4)


* SES score is a composite of Literacy, Income, Fuel, Cycle, Motor, TV, Cable, Mobile, and Mobile Expenditure.

### Lung Functions

The mean FVC, FEV1, PEFR, and FEV1/FVC (%) were lowest in the exposed group. The proportion of participants having FEV1/FVC (%) < 70% and FEV1% < 80% of predicted values were highest in the exposed group (Table 2). All lung function parameters were highest in the outdoor unexposed group. However, after adjusting for age, BMI, smoking status, SES, and years of exposure, FVC was not statistically different between the groups. PEFR remained statistically significantly lower in the exposed group compared to the indoor unexposed (mean difference (MD) = –0.75L, 95%CI = –1.36 to –0.15) (Table 3) and the outdoor unexposed group (MD = –0.81L, 95%CI = –1.44 to –0.19) (Table S2); FEV1/FVC (%) ratio was statistically significantly lower in the exposed group compared to the indoor unexposed (MD = –3.87, 95%CI = –6.77 to –0.96) (Table 3) and the outdoor unexposed (MD = –2.11, 95%CI = –5.14 to 0.92) groups (Table S2). Age was independently associated with lower FVC, FEV1, and FEV1/FVC (%). With every one-year increase in age there was a 20 ml decrease in FVC, 20 ml in FEV1, and 0.19 decrease in FEV1/FVC (%) ratio (Table S3), but there was no age effect seen for respiratory symptoms (Table S4).

**Table 2:** Comparison of Lung Function Between the Three Groups.


LUNG FUNCTION PARAMETERS	CEMENT EXPOSED N = 100	INDOOR UNEXPOSED N = 50	OUTDOOR UNEXPOSED N = 50

FVC (Mean, SD)	3.3 (0.7)	3.4 (0.6)	3.6 (0.6)

FEV1 (Mean, SD)	2.5 (0.6)	2.8 (0.6)	2.9 (0.6)

PEF (Mean, SD)	5.5 (1.7)	6.6 (1.7)	6.8 (1.7)

FEV1/FVC (%) (Mean, SD)	74.5 (8.7)	80.5 (6.9)	79.2 (8.9)

FEV1/FVC (%) < 70% N (%)	19 (20.2)	3 (6.3)	5 (10.6)

FEV1 % <80% of predicted FEV1% N (%)	67 (71.3)	31 (64.6)	26 (55.3)


* Normal FEV1/FVC (%) ratio should be at least is 70%. ** Normal value of FEV1% is more than or equal to 80%.

**Table 3:** Association of Cement Exposure with Lung Function and Respiratory Morbidity.


OUTCOMES	GROUPS	MEAN DIFFERENCE (95% CI), P VALUE	

		UNADJUSTED MD	ADJUSTED MD*

FVC (Liters)	Indoor un-exposed Outdoor un-exposed Cement-exposed	1 0.17 (–0.09, 0.44) –0.14 (–0.37, 0.08) **0.02**	1 0.16 (–0.09, 0.40) 0.06 (–0.16, 0.28) 0.45

FEV1 (Liters in 1 second)	Indoor un-exposed Outdoor un-exposed Cement-exposed	1 0.09 (–0.15, 0.34) –0.30 (–0.51, –0.09) **<0.001**	1 0.07 (–0.15, 0.29) –0.09 (–0.28, 0.11) 0.33

PEF (Liters/sec)	Indoor un-exposed Outdoor un-exposed Cement-exposed	1 0.18 (–0.51, 0.87) –1.11 (–1.70, –0.52) **<0.001**	1 0.58 (–0.62, 0.74) –0.75 (–1.36, –0.15) **0.01**

FEV1/FVC (%)	Indoor un-exposed Outdoor un-exposed Cement-exposed	1 –1.35(–4.73, 2.02) –5.99 (–8.91, –3.08) **<0.001**	1 –1.75 (–5.05, 1.54) –3.87 (–6.77, –0.96) **0.03**

		RATE RATIO** (95% CI), P VALUE	

Respiratory symptom score (0–13)	Indoor un-exposed Outdoor un-exposed Cement-exposed	1 1.13 (0.69, 1.85) 2.41 (1.57, 3.70) **<0.001**	1 1.11 (0.69, 1.79) 2.45 (1.63, 3.69) **<0.001**

		**ODDS RATIO (95% CI), P VALUE**	

FEV1/FVC (%) <70%	Indoor un-exposed Outdoor un-exposed Cement-exposed	1 1.79 (0.40, 7.94) 3.80 (1.06, 13.57) 0.07	1 2.31 (0.47, 11.32) 2.51 (0.64, 9.84) 0.41

FEV1 <80% of PredFEV1	Indoor un-exposed Outdoor un-exposed Cement-exposed	1 0.68 (0.30, 1.55) 1.36 (0.65, 2.86) 0.17	1 0.62 (0.26, 1.51) 0.95 (0.43, 2.12) 0.50

Any respiratory symptoms	Indoor un-exposed Outdoor un-exposed Cement-exposed	1 1.27 (0.58, 2.78) 7.21 (3.26, 15.93) **<0.001**	1 1.38 (0.60, 3.16) 7.38 (3.21, 17.00) **<0.001**

Any high-grade respiratory symptoms	Indoor un-exposed Outdoor un-exposed Cement-exposed	1 1 (0.36, 2.66) 3.55 (1.60, 7.87) **<0.001**	1 1.13 (0.41, 3.10) 3.46 (1.49, 8.00) **0.003**


* Adjusted for age, BMI, smoking, SES, and years of exposure. ** Zero-inflated Negative Binomial Regression. *** R2 for exposure to PEF = 0.04 and FEV1/FVC (%) = 0.03.

### Respiratory Symptoms

**Figure 2:**
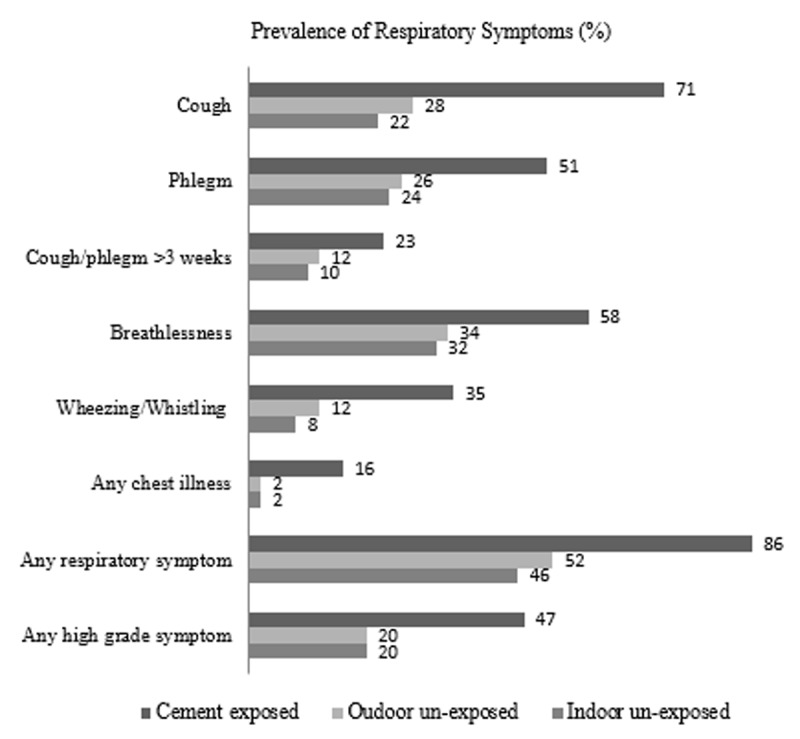
Prevalence of Respiratory Symptoms in the Three Study Groups.

All respiratory symptoms, including cough, phlegm, breathlessness, wheezing, and chest illness were higher in the exposed group. Of the exposed participants, 86% had “any respiratory symptoms” compared to only 50% in unexposed groups. Half (50%) of the exposed group had “any high-grade symptoms” against only 20% in unexposed groups (Figure 2 and Figure S1). The odds of having “any respiratory symptoms” were significantly higher in the exposed group compared to the indoor unexposed group (OR = 7.38, 95%CI = 3.21 to 17.0) (Table 3) and the outdoor unexposed (OR = 5.34, 95%CI = 2.27 to 12.59) groups (Table S2). Similarly, the odds of experiencing “any high-grade respiratory symptoms” were significantly higher in the cement-exposed group compared to the indoor unexposed (OR = 3.46, 95%CI = 1.49 to 8.00) (Table 3) and the outdoor unexposed (OR = 3.07, 95%CI = 1.30 to 7.24) group (Table S2). Respiratory symptom scores were significantly higher in the exposure group (rate ratio (RR) = 2.45, 95%CI = 1.63 to 3.69) (Table 3) compared to the indoor unexposed (RR = 2.21, 95%CI = 1.48 to 3.31) (Table S2) and outdoor unexposed groups. Respiratory symptom scores were negatively correlated with lung function (Pearson’s coefficient FEV1/FVC = –0.37, FEV1 = –0.30, FVC = –0.18), with higher respiratory symptoms indicating poorer lung function.

## Discussion

The higher burden of respiratory morbidity has been reported previously in workers exposed to cement dust but none of the studies earlier evaluated the respiratory health of informal workers exposed to cement dust. Informal workers exposed to cement in the present study had statistically significant lower lung function and higher respiratory symptoms when compared to unexposed informal workers. Informal workers engaged in outdoor work (not involving cement) had the best lung function when compared indoor unexposed and outdoor exposed groups. However, respiratory symptoms were least prevalent in indoor un-exposed informal workers. Increasing age was a significant factor in reduction of lung function across groups. To our knowledge this is the first study in India to report association of cement dust exposure with lung functions and respiratory morbidity among informal workers.

The control groups in the present study were chosen from contiguous geographical locations and somewhat similar sociodemographic characteristics. Since the exposed group was exposed to cement dust, environmental exposures, and engagement in strenuous physical labor, having an outdoor and indoor control group helped to adjust for environmental exposure as well as levels of physical activity, both of which have effects on lung function [22,23]. The concordance between self-reported respiratory symptoms and an objectively measured lung function (r=-0.37) is a proof of validity of the self-reported symptoms reported in this study. However, our study has certain limitations. Firstly, the ambient air pollution was not measured, and the occupation was considered as a proxy for exposure. Secondly, our sample was based on convenient sampling, which could have led to underestimation of respiratory symptoms and overestimation of lung function (selection bias and healthy worker effect). Thirdly, the symptoms recorded were self-reported symptoms, which may be affected by reporting bias, leading to over-reporting in the exposed and under-reporting in the unexposed groups. Fourthly, we did not measure post-bronchodilator lung function that would have helped in diagnosing COPD. Finally, this was a cross-sectional design hence causal associations cannot be established.

There were no studies available globally that measured lung function and respiratory morbidity among informal workers exposed to cement dust. Hence, we compared our study findings with studies performed among cement factory workers conducted in Sub-Saharan Africa (n = 9 studies), Middle East (n = 6), Europe (n = 3), and Asia (n = 3). None were reported from India. The choice of control groups, variables used of adjusted analyses, and the questionnaire used for capturing respiratory questionnaire were varied across studies. Some studies chose apparently healthy controls outside factory settings [7,10,24] and some within factory settings but with less exposure [5,6,8,9,11,12,13,14,24,25,26]. All of these studies (Table 4) showed a significantly lower lung function and higher respiratory symptoms in factory workers exposed to cement dust compared to controls, and the magnitude of difference varied depending upon the choice of control group and whether or not these differences were adjusted for confounders (Table 4). Studies that used healthy controls outside factory settings had demonstrated larger differences than studies with controls from the same setting but with lower exposure. A further two studies had used multiple controls similar to ours [12,26]. Choice of controls is an important design strategy to adjust for confounders.

**Table 4:** Comparison of lung functions and respiratory symptoms between exposed to cement vs. controls from previous literature.


AUTHOR, YEAR COUNTRY	EXPOSED	TYPE OF CONTROLS	UNADJUSTED DIFFERENCE BETWEEN EXPOSED AND UN-EXPOSED				UNADJUSTED DIFFERENCE IN SYMPTOM PERCENTAGE BETWEEN EXPOSED AND UN-EXPOSED			
	
			FVC ML	FEV1 ML	PEF ML/SEC	FEV1/FVC (%)	COUGH (%)	PHLEGM (%)	DYSPNEA (%)	WHEEZING (%)

*Present study*	***Informal workers*** ***exposed to cement***	***Indoor tailors*** ***Outdoor vegetable sellers***	***–100*** ***–300***	***–300*** ***–400***	***–1100*** ***–1300***	***–6*** ***–4.7***	***35*** ***33***	***18*** ***18***	***12*** ***11***	***27*** ***23***

Aweto, 2018 Nigeria [7]	Cement factory workers	Civil servants	–940	–830	–1060	–3.73	–	–	–	–

Tungu, 2014 Tanzania [12]	Cement factory workers	In 2002 Maintenance and administrationIn 2010 Mineral water production workers	–200 –80	–400 –100	– –	–10 –1	13.7 6	23.9 3.7	12.7 4.3	8.8 3.7

Tungu, 2015 Tanzania [27]	Cement production workers	Mineral water production workers					5	6	6	–1

Al-Neaimi, 2001 United Arab Emirates [5]	Factory supervisors, factory attendants, maintenance workers, machine operators	Workers attending the preventive medicine clinic to acquire a certificate of good health	–250	–400	–2560	–5.86	24.3	25.7	32.9	–

Rachiotis, 2018 Greece [10]	Cement production workers	White collar employees outside factory	% predicted +1	% predicted –1.5	–	–1.5	–	–	–	–

Ismaila, 2015 Nigeria [25]	Workers in cement production section	Workers in administrative section of factory	–	–	Given for combined 6.1l/s	–	–	–	–	–

Rafeemanesh, 2015 Iran [11]	High exposed workers from production and packing sections	Workers of the same factory without current and past exposure	% predicted –1.9	% predicted –2.7	–	–1	5	6	4	0

Aminian, 2014 Iran [6]	High exposed workers from production and packing sections	Less exposed office workers **Pre-shift** **Post-shift**	+210 +90	+190 +60	+170 +490	+0.45 +0.17	– –	– –	– –	– –

Meo, 2013 Pakistan[24]	Non-smoking cement mill workers	Clerical staff, shopkeepers, and salesmen <5 work experience >5 to <10 >10	–850 –1160 –740	–490 –630 –460	–738 –583 –1366	+5.7 +9.5 –4.3	– ––	– ––	– ––	– ––

Kakooei, 2012 Iran [8]	Male cement production line workers	Administrative sectionemployees.	–310	–310	–	–3	17.8	12	12.2	15.4

Zeleke, 2011 Ethiopia [26]2009	Cement factory cleaners and production workers	Security workers from both factories **2009** **Cleaners vs. production** **Cleaners vs. control** **Production vs. controls** **2010** **Cleaners vs. production** **Cleaners vs. control** **Production vs. controls**	+230 +210 –20 +220 +210 –10	+230 +130 –100 +170 +60 –110	– – – – – –	+1.02 –1.13 –2.15 +1.13 –2.34 –3.47	– – – – – –	– – – – – –	4.4 10.5 6.1 2.6 2.6 0	–0.5 18.7 19.2 13 34.2 21.2

Mbelambela, 2018 Kongo central province [9]	Cement factory workers (transportation, cleaning and production)	Less exposed- Administration, laboratory	–	–	–	–	13.3	8.5	2.5	2.2

Thepaksorn, 2013 Thailand [14]	Male roofing cement workers	Office workers (n=19) and subcontract workers	–	–	–	–	5.5		9.6	6.7

Ahmed, 2012 United Arab Emirates [13]	Workers exposed to cement dust	74 subjects not exposed to dust, from administration, finance and other department	–	–	–	–	14.4	13.5	6.9	–0.6


The mean lung function parameters in our exposed group were lower than the previously reported studies except for one, which reported a mean of FVC, FEV1, and PEF of 1.59L, 1.54L, and 4.04 L/s, respectively (Table S1) [7]. Similarly, the proportion with respiratory symptoms like cough, phlegm, breathlessness, and wheezing were higher (71%, 58%, 51%, and 35%) in our sample than previously reported. Based on this external comparison, we can hypothesize that informal workers were having a higher burden of respiratory illness compared to formal workers exposed to cement. This larger deficit among exposed informal workers could be due to underlying malnutrition and poor living conditions. However, a comparative study between formal and informal workers exposed to cement within Indian context is required to confirm this finding.

In this study we detected a difference in FVC that was smaller (small effect) than the differences in PEFR (moderate effect), and this could reflect the overall poorer lung capacity of informal workers across groups but with a higher level of obstructive air flow disease among the exposed workers. This could be an important finding that could inform the sample size calculation of future comparative studies.

Different questionnaires were used in previously reported studies to measure self-reported symptoms [5,7,8,14]. The most commonly used respiratory symptom questionnaires was from The British Medical Research Council (BMRC). Other questionnaires were those from the International Union against Tuberculosis and Lung Disease (IUATLD), the National Program of Silicosis Control, Iran, the Thai Thoracic Society, and The American Thoracic Society (ATS 1978). Irrespective of the questionnaire used, the prevalence of respiratory symptoms was higher when compared to the control groups. The proportion with cough varied from 15% to 70%, phlegm 15% to 45%, breathlessness 4% to 55%, and wheezing 5% to 67% among the exposed group, which was similar to the 69%, 50%, 58%, and 35%, respectively, observed in our study. However, none reported a summary score of any symptoms and high-grade symptoms.

Our study findings are important, as informal workers are often ignored in surveys and regulatory acts that safeguard the rights and safety of workers. In India, since a large percentage (92%) of workers are involved in informal employment [16], these populations are vulnerable in several ways; they are usually migrant workers with no social security cover, poor access to health care, and not covered under the Factories Act. They are daily wage earners by default and only the fittest continue to work. Hence, documenting the ill effects of hazardous occupations is crucial to obtain unbiased estimates of the burden of risk factors and health outcomes. Further amendment of existing policies related to workplace safety that cover informal workers are urgently required. Policy changes should make employers of contractual laborers responsible for worker’s safety, such as provision of protective gear to the exposed workers and making its use mandatory.

To conclude, our study demonstrated that informal workers exposed to cement dust showed poorer respiratory health when compared to those who are not exposed to cement dust. Impactful public health policies inclusive of informal workers are urgently needed to protect India’s workforce.

## Additional File

The additional file for this article can be found as follows:

10.5334/aogh.4089.s1 Supplementary Material. Includes a box describing the scoring of respiratory symptoms, four tables (Tables S1–S4), and one Figure (Figure S1).
