# Supplementary material for: Lung Function and Respiratory Morbidity Among Informal Workers Exposed to Cement Dust: A Comparative Cross-Sectional Study
Source: Ann Glob Health. 2023 Jul 4;89(1):47. doi: 10.5334/aogh.4089 (PMC10327864; doi:10.5334/aogh.4089)

Supplementary

BOX: Scoring of respiratory questionnaire

Any respiratory symptom: Yes/No

Yes, if any of the six main symptoms namely ‘cough’, ‘phlegm’, ‘breathlessness’, ‘wheezing/whistling’, ‘wheezing/whistling with breathlessness’, and ‘woken up due to shortness of breath’ were answered ‘yes’

High grade symptom: Yes/No

If “Yes” to any of the following

(1) cough on most days for as much as 3 months each year

(2) bring up phlegm on most days for as much as 3 months each year,

(3) In the past 3 years had a period of (increased) cough and phlegm lasting for 3 weeks or more

(4) have to stop for breath when walking at your own pace on level ground,

(5) breathing not normal between attacks of breath with wheezing

(6) any chest illness kept away participant from his usual work as long as 7days

Respiratory symptom score: 0-13 higher scores indicating higher morbidity

A weighted composite symptom score was created using all the respiratory symptoms. The weighting was as follows; for cough and phlegm symptoms, “0” score was given if participant was not having any cough and phlegm symptoms, “1” if participant was having cough or phlegm either during day or night but not as much as 3 months in a year, “2” if participant was having cough or phlegm both during day and night but not as much as 3 months in a year, “3” if participant was having cough or phlegm either during day or night and as much as 3 months in a year; for breathlessness “0” if he had no breathlessness symptom, “1” if he had shortness of breath when hurrying on level ground, “2” if he had shortness of breath walking with other people of your own age on level ground and “3” if

he had to stop for breath when walking at your own pace on level ground; “1” grade if wheezing/whistling in last 12 months; “1” if he ever had wheezing/whistling with breathlessness, “2” if he was not normal between attacks; “1” grade if he was woken due to shortness of breath in last 12 months.

Table S1: Comparison of respiratory morbidity of present study with previous literature

**Study, year (sample size)**	**Country**	**FVC (Mean, SD)**	**FEV1 (Mean, SD)**	**PEF (Mean, SD)**	**FEV1/FVC (%) (Mean, SD)**	**FEV1/ FVC < 70% (%)**	**FEV1 % <80% of predicted FEV1% (%)**
**Present study**		3.3 (0.7)	2.5 (0.6)	5.5 (1.7)	74.5 (8.7)	20.2	71.3
**Aweto, 2018 (70)**	Nigeria	1.59 (0.64)	1.54 (0.63)	4.04 (1.88)	93.07 (6.28)	-	-
**Tungu, 2014 (115)**	Tanzania	3.30 (0.5)	2.60 (0.5)	-	77 (0.6)	22.6	
**Al-Neaimi, 2001 (67)**	United Arab Emirates	3.17 (SE 0.08)	2.59 (0.07)	5.19 (0.23)	81.78 (1.11)	-	-
**Rachiotis, 2018 (137)**	Greece	-	-	-	-	4.4	13.9
**Rafeemanesh, 2015 (100)**	Iran	-	-	-	-	-	-
**Aminian, 2014 (100)**<br>**Pre-shift**<br>**Post-shift**	Iran	4.73 (0.69)<br>4.66 (0.68)	3.78 (0.62)<br>3.68 (0.60)	9.51 (1.50)<br>9.05 (1.42)	79.79 (6.18)<br>79.09 (6.16)	-	-
**Meo, 2013**<br>**<5 work experience-(10)**<br>**>5 to <10(10)**<br>**>10- 30**	Pakistan	3.40 (SEM 0.33)<br>3.11 (0.22)<br>3.13 (0.14)	2.86 (0.29)<br>2.61 (0.22)<br>2.41 (0.11)	5.22 (0.59)<br>5.31 (0.74)<br>4.72 (0.41)	84.8 (3.29)<br>85.50 (5.96)<br>79.00 (3.01)	-	-
**Kakooei, 2012 (94)**	Iran	3.86	3.10	-	79	40.4	48.9
**Zeleke, 2011**<br>**2009**<br>**Cleaners (38)**<br>**Production (33)**<br>**2010**<br>**Cleaners (38)**<br>**Production (33)**	Ethiopia	4.05 (0.69)<br>3.82(0.58)<br><br>4.01(0.65)<br>3.79(0.53)	3.46(0.67)<br>3.23(0.55)<br><br>3.36(0.65)<br>3.19(0.57)	-	85.19(6.3)<br>84.17(5.96)<br><br>83.49(7.5)<br>82.36(6.58)	-	

Table S2: Association of cement exposure with lung function and respiratory morbidity (Base group - Outdoor un-exposed)

**Outcomes**	**Groups**	**Mean Difference (95% CI), P value**	
		**Unadjusted MD**	**Adjusted* MD**
**FVC (Liters)**	Outdoor un-exposed<br>Indoor un-exposed<br>Cement exposed	1<br>-0.17 (-0.44, 0.09)<br>-0.32 (--0.55, -0.09)<br>**0.02**	1<br>-0.16 (-0.40, 0.09)<br>-0.01 (-0.33, 0.13)<br>0.45
**FEV1 (Litres in 1 second)**	Outdoor un-exposed<br>Indoor un-exposed<br>Cement exposed	1<br>-0.09 (-0.34, 0.15)<br>-0.39 (-0.61, -0.18)<br>**<0.001**	1<br>0.07 (-0.29, 0.15)<br>-0.15 (-0.35, 0.05)<br>0.33
**PEF (Litres/sec)**	Outdoor un-exposed<br>Indoor un-exposed<br>Cement exposed	1<br>-0.18 (-0.87, 0.51)<br>1.29 (-1.88, -0.69)<br>**<0.001**	1<br>-0.58 (-0.74, 0.62)<br>-0.81 (-1.44, -0.19)<br>**0.01**
**FEV1/FVC**	Outdoor un-exposed<br>Indoor un-exposed<br>Cement exposed	1<br>1.35(-2.02, 4.73)<br>-4.64 (-7.57, -1.70)<br>**<0.001**	1<br>1.75 (-1.54, 5.05)<br>-2.11 (-5.14, 0.92)<br>**0.03**
		**Rate ratio** (95% CI), P value**	
**Respiratory symptom score (0-13)**	Outdoor un-exposed<br>Indoor un-exposed<br>Cement exposed	1<br>0.89 (0.54, 1.46)<br>2.14 (1.40, 3.28)<br>**<0.001**	1<br>0.90 (0.56, 1.46)<br>2.21 (1.48, 3.31)<br>**<0.001**
		**Odds Ratio (95% CI), P value**	
**FEV1/FVC <0.70**	Outdoor un-exposed<br>Indoor un-exposed<br>Cement exposed	1<br>0.56 (0.13, 2.49)<br>2.13 (0.74, 6.11)<br>0.07	1<br>0.43 (0.09, 2.14)<br>1.09 (0.33, 3.61)<br>0.41
**FEV1 <80% of PredFEV1**	Outdoor un-exposed<br>Indoor un-exposed<br>Cement exposed	1<br>1.47 (0.65, 3.36)<br>2.00 (0.97, 4.15)<br>0.17	1<br>1.61 (0.66, 3.92)<br>1.53 (0.67, 3.48)<br>0.50
**Any respiratory symptom**	Outdoor un-exposed<br>Indoor un-exposed<br>Cement exposed	1<br>0.79 (0.36, 1.72)<br>5.67 (2.57, 12.52)<br>**<0.001**	1<br>0.72 (0.32, 1.65)<br>5.34 (2.27, 12.59)<br>**<0.001**
**Any high grade respiratory symptom**	Outdoor un-exposed<br>Indoor un-exposed<br>Cement exposed	1<br>1 (0.38, 2.66)<br>3.12 (1.60, 7.87)<br>**<0.001**	1<br>0.89 (0.33, 2.45)<br>3.07 (1.30, 7.24)<br>**0.003**

Adjusted for age, BMI, smoking, SES, and years of exposure
***** Zero-inflated Negative Binomial Regression***

Table S3: Risk factors of Lung functions

Mean difference	FVC (Litres)		FEV1(Litres in 1 second)		PEF(Litres/sec)		FEV1/FVC	
	**Un-adjusted (95%CI), P value**	**Adjusted (95%CI), P value**	**Un-adjusted (95%CI), P value**	**Adjusted (95%CI), P value**	**Un-adjusted (95%CI), P value**	**Adjusted (95%CI), P value**	**Un-adjusted (95%CI), P value**	**Adjusted (95%CI), P value**
**Exposure**<br>**Indoor**<br>**Outdoor**<br>**Cement**	1<br>0.17 (-0.09, 0.44)<br>-0.14 (-0.37, 0.08)<br>**0.02**	1<br>0.16 (-0.09, 0.40)<br>0.06 (-0.16, 0.28)<br>0.45	1<br>0.09 (-0.15, 0.34)<br>-0.30 (-0.51, -0.09)<br>**<0.001**	1<br>0.07 (-0.15, 0.29)<br>-0.09 (-0.28, 0.11)<br>0.33	1<br>0.18 (-0.51, 0.87)<br>-1.11 (-1.70, -0.52)<br>**<0.001**	1<br>0.58 (-0.62, 0.74)<br>-0.75 (-1.36, -0.15)<br>**0.01**	1<br>-1.35(-4.73, 2.02)<br>-5.99 (-8.91, -3.08)<br>**<0.001**	1<br>-1.75 (-5.05, 1.54)<br>-3.87 (-6.77, -0.96)<br>**0.03**
**Age (yrs.)**	-0.03 (-0.03, -0.02)<br>**<0.001**	-0.02 (-0.04, -0.01)<br>**<0.001**	-0.03 (-0.04, -0.02)<br>**<0.001**	-0.02 (-0.03, -0.012)<br>**<0.001**	-0.05 (-0.07, -0.03)<br>**<0.01**	-0.03 (-0.06, 0.01)<br>0.12	-0.30 (-0.40, -0.21)<br>**<0.001**	-0.19 (-0.35, -0.02)<br>**0.03**
**BMI (kg/m^2)**	-0.04 (-0.06, -0.01)<br>**0.01**	-0.03 (-0.02, 0.002)<br>0.07	-0.04 (-0.06, -0.01)<br>**0.01**	-0.03 (-0.05, -0.001)<br>**0.04**	-0.001 (-0.08, 0.76)<br>0.97	0.03 (-0.05, 0.10)<br>0.51	-0.24 (-0.061, 0.14)<br>0.21	-0.12 (-0.49, 0.24)<br>0.50
**Smoking**<br>**Never**<br>**Former**<br>**Current**	1<br>-0.05 (-0.5, 0.4)<br>-0.01 (-0.21, 0.2)<br>0.98	1<br>0.06 (-0.35, 0.47)<br>-0.04 (-0.26, 0.15)<br>0.87	1<br>-0.10 (-0.53, 0.33)<br>-0.04 (-0.24, 0.16)<br>0.85	1<br>0.03 (-0.34, 0.39)<br>-0.07 (-0.24, 0.10)<br>0.72	1<br>0.31 (-0.91, 1.54)<br>-0.08 (-0.64, 0.47)<br>0.82	1<br>0.62 (-0.51, 1.75)<br>-0.15 (-0.67, 0.37)<br>0.42	1<br>-2.46 (-8.48, 3.50)<br>-0.85 (-3.57, 1.86)<br>0.63	1<br>-0.84 (-6.31, 4.63)<br>-0.85 (-3.37, 1.66)<br>0.79
**SES score**	0.08 (0.02, 0.13<br>**0.01**	0.04 (-0.02, 0.10)<br>0.17	0.10 (0.05, 0.16)<br>**<0.001**	0.06 (0.004, 0.11<br>**0.04**	0.30 (0.16, 0.45)<br>**<0.001**	0.15 (-0.01, 0.31)<br>0.07	1.34 (0.62, 2.07)<br>**<0.001**	0.72 (-0.05, 1.49)<br>0.07
**Years of working**	-0.02 (-0.03, -0.01)<br>**<0.001**	0.002 (-0.01, 0.01)<br>0.77	-0.03 (-0.03, -.02)<br>**<0.001**	-0.0004 (-0.01, 0.01)<br>0.95	-0.05 (-0.07, -0.03)<br>**<0.001**	-0.02 (-0.05, 0.02)<br>0.33	-0.27 (-0.38, -0.16)<br>**<0.001**	-0.06 (-0.23, 0.12)<br>0.53

Adjusted for age, BMI, smoking, SES, and years of exposure

Table S4: Risk factors of respiratory symptoms

	**Any respiratory symptom**		**Any high grade respiratory symptom**		**Respiratory symptom score**	
	**Un-adjusted OR (95% CI), P value**	**Adjusted OR (95% CI), P value**	**Un-adjusted OR (95% CI), P value**	**Adjusted OR (95% CI) , P value**	**Un-adjusted RR** (95% CI) , P value**	**Adjusted RR** (95% CI) , P value**
**Exposure**<br>**Indoor**<br>**Outdoor**<br>**Cement**	1<br>1.27 (0.58, 2.78)<br>7.21 (3.26, 15.93)<br>**<0.001**	1<br>1.38 (0.60, 3.16)<br>7.38 (3.21, 17.00)<br>**<0.001**	1<br>1 (0.36, 2.66)<br>3.55 (1.60, 7.87)<br>**<0.001**	1<br>1.13 (0.41, 3.10)<br>3.46 (1.49, 8.00)<br>**0.003**	1<br>1.13 (0.69, 1.85)<br>2.41 (1.57, 3.70)<br>**<0.001**	1<br>1.11 (0.69, 1.79)<br>2.45 (1.63, 3.69)<br>**<0.001**
**Age (yrs.)**	1.03 (1, 1.05)<br>0.07	1 (0.95, 1.04)<br>0.86	1.03 (1, 1.05)<br>**0.03**	1.01 (0.97, 1.06)<br>0.50	1.01 (1, 1.03)<br>**0.06**	1 (0.98, 1.02)<br>0.70
**BMI (kg/m^2)**	1.0 (0.92, 1.10)<br>0.97	0.98 (0.89, 1.09)<br>0.74	1.04 (0.95, 1.13)<br>0.44	1.01 (0.91, 1.12)<br>0.86	1.0 (0.96, 1.05)<br>0.85	0.99 (0.95, 1.04)<br>0.77
**Smoking**<br>**Never**<br>**Former**<br>**Current**	1<br>1.61 (0.32, 8.10)<br>0.82 (0.44, 1.54)<br>0.66	1<br>1.70 (0.30, 9.72)<br>0.80 (0.39, 1.65)<br>0.65	1<br>0.49 (0.10, 2.44)<br>0.67 (0.35, 1.28)<br>0.36	1<br>0.45 (0.08, 2.46)<br>0.66 (0.33, 1.33)<br>0.38	1<br>0.55 (0.29, 1.04)<br>0.80 (0.57, 1.14)<br>0.33	1<br>0.64 (0.32, 1.28)<br>0.70 (0.51, 0.98)<br>0.28
**SES score**	0.84 (0.70, 1)<br>0.06	1 (0.81, 1.24)<br>0.98	0.88 (0.73, 1.06)<br>0.17	1.04 (0.83, 1.29)<br>0.75	0.89 (0.81, 0.98)<br>**0.02**	0.92 (0.83, 1.02)<br>0.13
**Years of working**	1.02 (1, 1.06)<br>0.10	1.02 (0.97, 1.06)<br>0.53	1.03 (1, 1.05)<br>0.07	1.01 (0.96, 1.05)<br>0.75	1.01 (0.99, 1.02)<br>0.37	0.99 (0.97, 1.01)<br>0.37

Adjusted for age, BMI, smoking, SES, and years of exposure

***** Zero-inflated Negative Binomial Regression***

Figure S1: Prevalence of all Respiratory Symptoms (as recorded using BMRC-questionnaire)

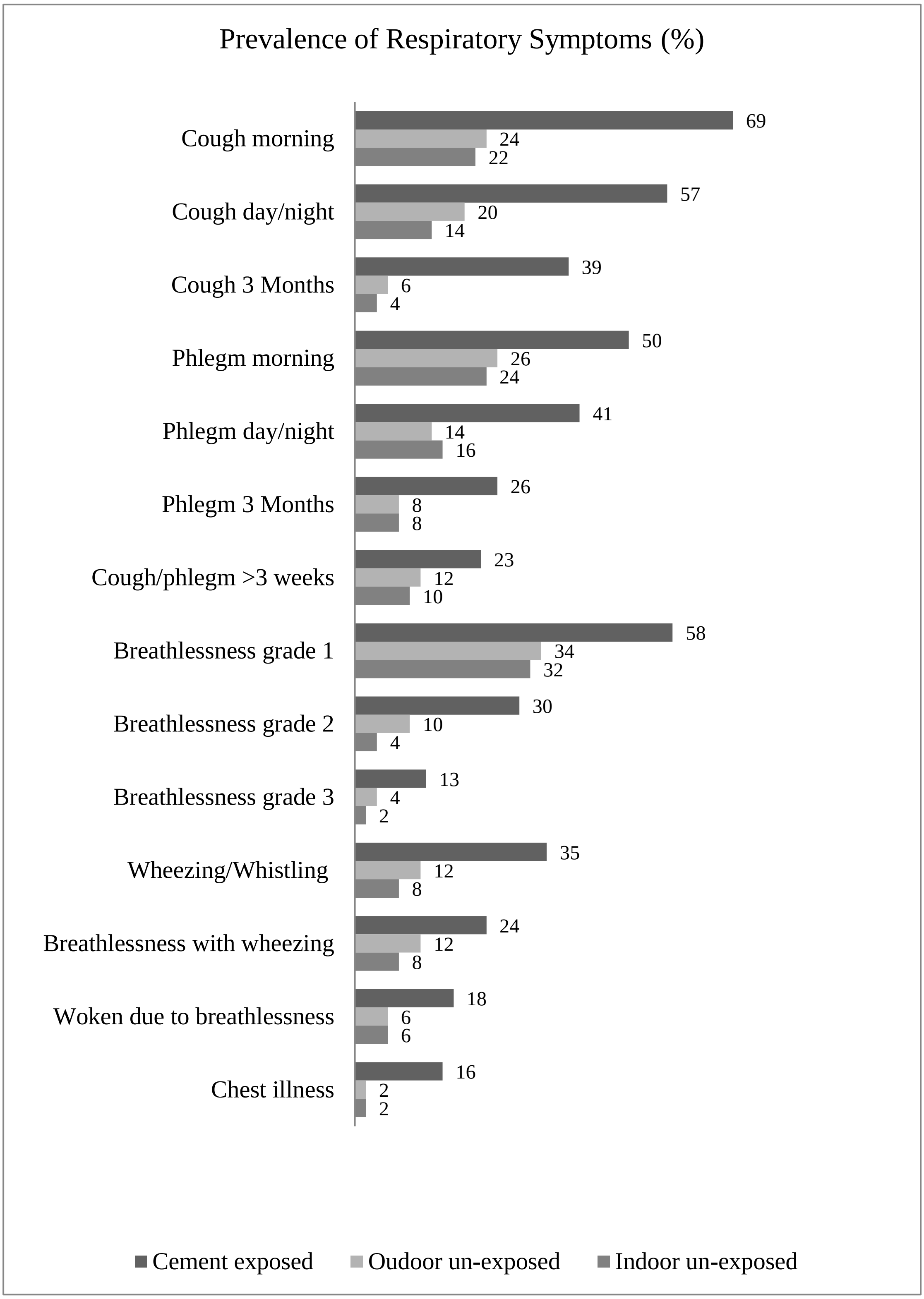

Supplement: Supplementary Material. — Includes a box describing the scoring of respiratory symptoms, four tables (Tables S1–S4), and one Figure (Figure S1). [file agh-89-1-4089-s1.pdf]
